# NeoVault: empowering neonatal research through a neonate data hub

**DOI:** 10.1186/s12887-024-05276-y

**Published:** 2024-11-30

**Authors:** Janet Pigueiras-del-Real, Angel Ruiz-Zafra, Isabel Benavente-Fernández, Simón P. Lubián-López, Syed Adil Hussain Shah, Syed Taimoor Hussain Shah, Lionel C. Gontard

**Affiliations:** 1 Department of Condensed Matter Physics, University of Cádiz, Cádiz, 11510 Spain; 2 Department of Software Engineering, University of Granada, Granada, 18071 Spain; 3 Division of Neonatology, Department of Paediatrics, Puerta del Mar University Hospital, Cádiz, 11019 Spain; 4 Research Unit, Biomedical Research and Innovation Institute of Cádiz (INiBICA), Cádiz, 11019 Spain; 5 Area of Paediatrics, Department of Child and Mother Health and Radiology - Medical School, University of Cádiz, Cádiz, 11003 Spain; 6 Department of Research and Development (R&D), GPI SpA, Trento, 38123 Italy; 7 PolitoBIOMed Lab, Department of Mechanical and Aerospace Engineering, Politecnico di Torino, Turin, 10129 Italy; 8 IMEYMAT, University of Cadiz, Cádiz, 11510 Spain

**Keywords:** Preterm, Data hub, Pose tracking, API, Physiological, Services, Neovault

## Abstract

**Background:**

Stability during early postnatal life in preterm infants is related to better outcomes. Although vital signs are monitored continuously in Neonatal Intensive Care Unites, this monitoring does not include all physiological parameters nor data such as movement patterns. Although there are scattered sources of data, there is no centralized data hub for neonates information.

**Results:**

We have created the first neonate data hub for easy and interactive access to upload or download postural, physiological, and medical data of neonates: NeoVault. NeoVault is a platform that provides access to information through two interfaces: *1)* via a Web interface (designed for medical personnel, data scientists, researchers); and *2)* via a RESTful API (Application Programming Interfaces) -designed for developers-, aiming to integrate access to information into third-party applications. The web access allows searching and filtering according to specific parameters, visualization of data through graphs and images, and generation of datasets in CSV format. Access through the RESTful API is described in OpenAPI, enabling access to information from any device, facilitating it in an interoperable format. Currently, it contains nearly 800,000 postural records and 3.000 physiological data entries. The physiological and postural data stored for each neonate in NeoVault are collected through the NRP (*Neonates Recording Platform*) tool, which allows for the automatic and reliable collection of data.

**Conclusion:**

NeoVault is an open platform for simple access to postural, physiological, and medical data of neonates that can be utilized by researchers, data scientists, medical personnel, and programmers. It enables integration into third-party applications and the generation of customized datasets.

## Background

Preterm infants are babies born before the 37^th^ week of gestation. Due to their immature development they can face an increased risk of experiencing respiratory problems (e.g bronchopulmonary dysplasia), cardiac issues, infections, and neurodevelopmental disorders [1–4].

Due to their physical conditions, they are admitted to the Neonatal Intensive Care Unit (*NICU*), where the clinical evaluation of the baby’s health and constant monitoring of vital signs are carried out. This health assessment is most often conducted through visual observation of the infants’ behavioral traits (movements, facial expressions, crying), neuroimaging exploration, and cardio-respiratory and ECG monitoring by neonatologists [5, 6].

The medical information collected from the clinical analysis of preterm infants is often documented in notes or reports crafted by specialists. Additionally, in certain instances, is stored in private platforms or storage systems (such as in the case of medical images) accessible only to designated healthcare professionals, ensuring privacy of the patient´s information.

In recent times, there has been a rising interest in exploring the collection and subsequent analysis of video and audio data as non-invasive methods for information gathering. This data, upon thorough examination, has the potential to improve the clinical monitoring of a neonate’s health and the assessments conducted in a NICU [5, 7–11].

The integration of this medical information into databases (gathered by collection tools or generated in hospitals) significantly streamlines the process for medical staff when reviewing records. This technological advancement not only enhances efficiency but also serves as a valuable tool in gaining insights into the effectiveness of various treatments. By centralizing and organizing the data, the database provides medical staff with a comprehensive and accessible platform for thorough record analysis. This, in turn, contributes to a more informed understanding of the efficacy of different treatment modalities, fostering continuous improvement in patient care and outcomes [12–14].

Public medical databases that house information about preterm infants play an essential role in scientific research, healthcare enhancement, early detection of health issues, and the innovation of new therapies and medications. Beyond that, these databases serve as invaluable assets for the education of healthcare professionals, students, and scientists. They actively promote collaboration among researchers and healthcare professionals, propelling technological advancement by creating a conducive environment for the testing and development of cutting-edge data analysis tools and information technologies in the realm of healthcare [15].

To the best of the authors’ knowledge, there is a lack of publicly accessible databases specifically designed for the study of neonates. This scarcity is primarily attributed to privacy and security considerations that place constraints on obtaining ethical approval [5]. Nevertheless, it is noteworthy to mention some data-sets and database that could be found:

1. *BabyPose*: it is a data-set, encompassing data on 12 limb-joint locations and depth images related to the movement of neonates [15, 16].
2. *MIAdataset*: it consists in the states vector, along with the corresponding timestamp, derived from depth measurements of a preterm infant. It contains a timeline of 16 different states in which the infant under examination was in. To obtain this dataset, you have to complete, sign and return a form that you can find in the web page where is located the information of the dataset. After that, you will receive the credentials to download it. Note that the data-set is available only for research purposes [17].
3. *Preterm Clinical Network (PCN)*: a web-based systematic method for collecting data concerning the care of women at risk of preterm birth. Notably, it incorporates a registry of children born to women at risk, who have undergone specialized preterm surveillance and and may have received preterm interventions, whether born prematurely or not. However, it is essential to note that it is not specifically a database providing neonatal-specific information, and direct access to the data is not feasible; interested parties can obtain it by making a request to the corresponding author [12].
4. *MMSdataset*: the Multi-Modal Stimulations data-set contains preterm infants data including: gestational age, chronological age, corrected gestational age, sex, birth weight, birth length, birth occipitofrontal circumference (OFC), APGAR at 1-min, and APGAR at 5-min, pre-post intervention (5 days) changes of weight, length, INFANIB (Infant Neurological International Battery) and NIPS (Neonatal Infant Pain Scale) [18, 19].

The aforementioned databases and datasets exhibit certain constraints that merit consideration: *1)* the issue of direct access arises, as some databases demand the initiation of a formal access request process. This can introduce delays and procedural hurdles in obtaining the required data; *2)* the absence of a user-friendly website interface is notable. A streamlined and intuitive interface can significantly enhance the user experience, particularly in terms of exporting data, reformatting information, or seamlessly integrating new data into the existing system. This aspect becomes important in ensuring efficient utilization and accessibility for diverse users; *3)* a noteworthy limitation pertains to the scope of information within these databases, particularly concerning body pose reference points. The available data-sets offer insights into a limited set of reference points, potentially constraining comprehensive analyses or applications requiring a more extensive array of pose-related data.

Moreover, a crucial observation is the absence of databases containing detailed information on the physiological parameters of neonates. This gap in the available resources underscores a potential limitation in comprehensive research and analysis focused on understanding and addressing the unique healthcare needs of preterm infants. As such, tackling these constraints could significantly contribute to the advancement of a research and medical care in the neonatal domain.

To overcome these limitations, we have developed NeoVault the first neonate data hub. NeoVault provides public access to a comprehensive collection of perinatal and neonatal data. Besides it goes beyond traditional databases by encompassing vital physiological parameters and data of 33 reference points (landmarks), which are crucial for understanding preterm infant body pose. The different data sources currently considered in this first version of NeoVault are shown in Table 1.

**Table 1:** Data sources collected and available in NeoVault

Parameter	Description	Expected values	Justification for inclusion in NeoVault (medical relevance)
**Sex**	Biological sex at birth	Boy, Girl, Both	It is essential for improving the accuracy in risk assessment, personalizing treatments, and enhancing short and long-term outcomes, considering the biological differences that may influence the health and development of newborns [20, 21]
**Gestational Age**	Age of a baby during pregnancy	[25,36] Weeks	It is essential for assessing risk, planning interventions, predicting long-term development, and improving neonatal care protocols [22, 23]
**Birth Size**	Baby size, measuring from head to toe	[20,50] Centimeters	It is fundamental for assessing the health of the newborn and predicting short- and long-term risks. This variable allows for the identification of at-risk neonates, personalization of medical care, improved monitoring of development, and ultimately, optimization of both immediate and future health outcomes [24]
**Birth Head Circumference**	Head circumference of the baby	[15,45] Centimeters	This variable not only provides critical information about fetal growth but also helps predict short- and long-term risks, allowing for timely and appropriate interventions to improve the health outcomes of the newborn [25, 26]
**Birth Weight**	Newborn weight	[0-$\infty$] Grams	It is a key indicator for assessing the health of the newborn and predicting short- and long-term risks [22]
**APGAR1 and APGAR5**	Quick assessment performed on a newborn at 1 minute and 5 minutes	[0,10]	The APGAR score is useful for the assessment and initial management of the newborn’s health. Its use not only provides critical information about the newborn’s condition at birth but also allows for monitoring their development and health over time, thereby contributing to improved outcomes in neonatal care [27–29]
**CRIB**	Clinical Risk Index for Babies	[0,23]	The CRIB score helps identify newborns at risk and provides valuable information to optimize care and improve long-term health outcomes [30, 31]
**Brain Damage**	Brain damage or neurological damage	Yes/No	Recording neonatal brain damage in a database is essential for tracking development, guiding personalized care, and improving long-term outcomes. It helps link brain injury with clinical factors, enabling better treatment and research into neurological development [32]
**Vital Signs**	SP02 (Oxygen Saturation) and Heart Rate (Times of heart beats per minute - BPM-)	[0,100] for SP02 and [,220] for Heart Rate	The monitoring of vital signs is essential for the continuous assessment and proper care of newborns. This data not only facilitates the early detection of health issues but is also crucial for personalizing treatment, improving clinical outcomes, and contributing to the development of best practices in neonatal care [33, 34]
**Pose Estimation**	A total of 33 body points, each with x, y, and z values (spatial position)	[0,1] for x and y [−1,1] for z (normalized values)	The study of neonatal movement patterns (pose tracking), along with other clinical data (weight, gestational age, physiological parameters), can be used to assess motor development and facilitate the early identification of neurological or motor anomalies [32, 35]

At the forefront of artificial intelligence, NeoVault functions as a platform for training cutting-edge AI models, facilitating the automating early detection of health problems (such as neurological and motor [35–38]) and holding the potential to revolutionize the efficiency and accuracy of neonatal care. Noteworthy is NeoVault ’s user-friendly interface, enabling seamless navigation through complex queries, data mining, and visualization, positioning it as an indispensable tool in the realm of preterm infants’ healthcare and research.

Furthermore, NeoVault includes a high-level API (*Application Programming Interface*) implemented through a RESTful style [39] to provide a set of web services than can be used by developers/programmers to access data automatically or integrate data access in third-party applications.

In summary, NeoVault is a proposal for promoting open science by the collection of large and rich standardized datasets in the field of neonatal care. The primary focus of NeoVault is to provide a web for compiling neonatal information of clinical interest, accessible for reading and writing by both medical personnel and researchers. A data hub like NeoVault can be useful not only for easily visualizing and searching for information but also for providing a compilation of different measurands of clinical interest that often are found scattered in different data-bases, that are rarely stored in clinical settings, or that until recently have not been recorded as they depend on the subjective observations done by medical staff. Numerous studies have shown that combining data from various sources can be beneficial for evaluating the physical condition of neonates [40], predicting risks [41], or associating movement patterns with motor development or the early identification of neurological or motor anomalies [32]. NeoVault not only provides all this information, but its access is also interactive and user-friendly, allowing users to obtain organized and structured data ready for analysis, without the need to worry about data cleaning or curation.

## Construction and content

NeoVault is a platform for interactive, real-time access, from any device, to physiological, medical, and postural information of neonates. NeoVault is hosted on https://conversational.ugr.es/neovault/. NeoVault follows a client-server architecture implemented through a Service-Oriented Architecture (SOA) philosophy [42].

In the server-side (also known as *backend*) of NeoVault, a set of web services are implemented to provide functionality: registering new neonates, searching by filters, listing all neonates, obtaining physiological parameters, generating datasets, etc. These services are implemented in Python using the Flask framework^1^. Additionally, other libraries are used for data processing and other functionalities such as Pandas^2^, Numpy^3^, os, zipfile, and shutil. MySQL is used as the database management system, and its interaction with the services is done through the SQLAlchemy library^4^.

One of the functionalities implemented in the backend, in addition to information management, is the ability to generate an animated image (GIF) from a set of postural data. This is done for each package of postural data uploaded for the neonate, to visualize it illustratively later on. This functionality is implemented using the MediaPipe^5^, Matplotlib, and ImageMagick libraries.

On the other hand, NeoVault’s front-end utilizes native HTML5, CSS, and Javascript technologies. Additional libraries are used for design (CSS), such as Bootstrap^6^ and Fontawesome, and for functionality (Javascript), such as JQuery^7^ and Highcharts^8^.

All the information in NeoVault is accessible through two different interfaces:

1. *Web Interface*. Through the options on the website, users can list available data, filter the information according to medical parameters as well as by date, and generate custom datasets. These datasets are stored in CSV files, so they can be easily exploited by doctors and data scientists through data processing software such as Excel, Jupyter, etc.
2. *API* (Application Programming Interface). A RESTfull API is provided for programmers to access information automatically, aiming to integrate medical data in real-time into other types of systems. The API specification is described in OpenAPI, accessible from the same website through a dedicated webpage. All information returned by the services is represented in JSON format to ensure operability and enable access to the information from any device and platform.

NeoVault is a platform that has been created from scratch, aiming to expand its dataset over time. Currently, there are data registered for 11 neonates (8 boys and 3 girls) including the following medical indicators: sex, gestational age, birth size, head circumference at birth, birth weight, Apgar 1 and Apgar 5 tests, CRIB (*Clinical Risk Index for Babies*), and whether the neonate has brain injury or not assessed by clinicians through brain ultrasonography.

For each neonate, there are records at different time points regarding their physiological parameters (heart rate and oxygen saturation) and postural data. In total, as of March 2024, NeoVault has approximately 3000 physiological records (each containing heart rate and oxygen saturation) and close to 800.000 postural records, where each record stores information for 33 body points (nose, left elbow, various parts of the eyes, etc.) in three spatial coordinates (*x, y, z*).

**Fig. 1:**
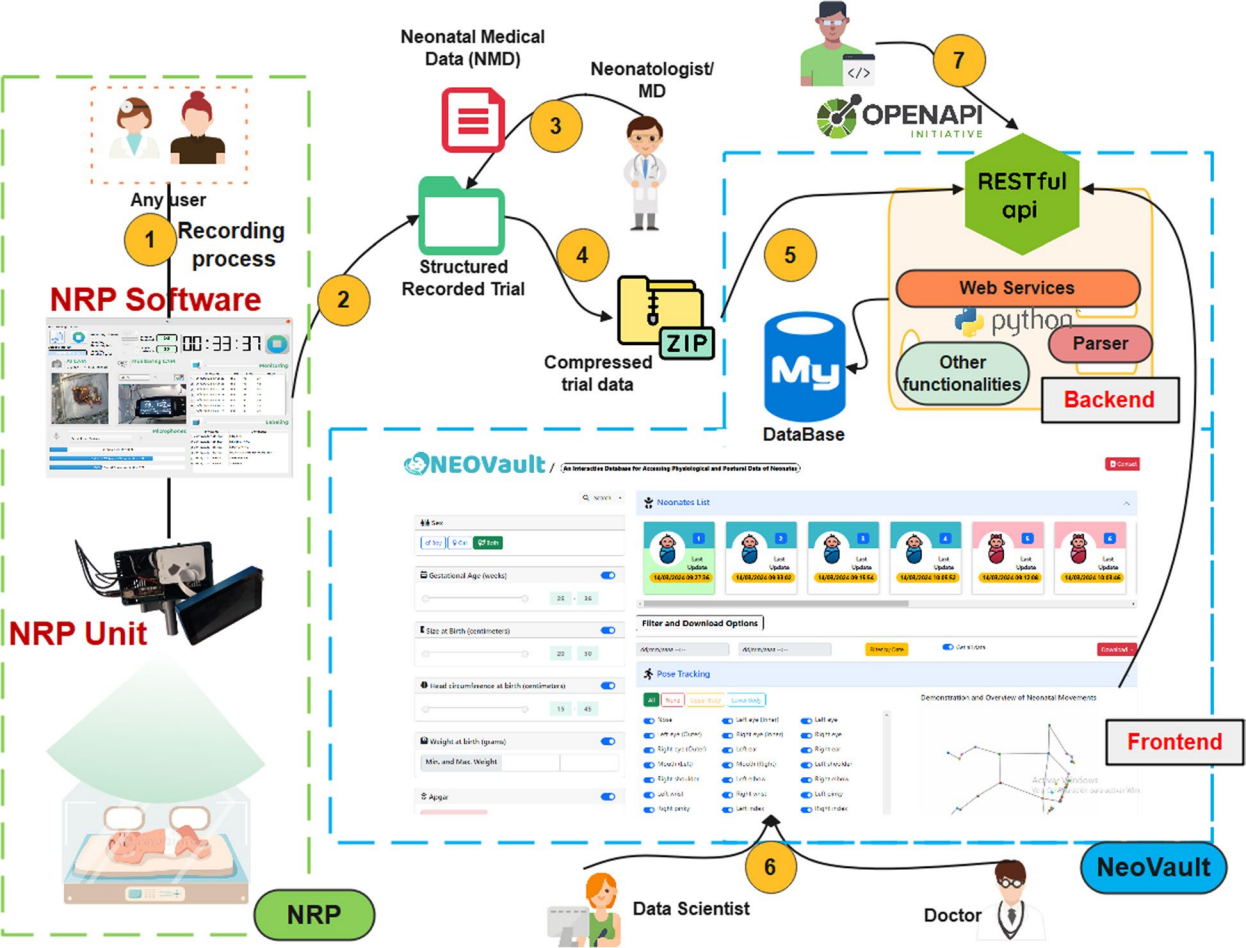
NeoVault architecture and workflow

The Fig. 1 shows the architecture and data flow of NeoVault.

The data have been collected using the *Neonate Recording Platform (NRP)* [8], which was deployed at Puerta del Mar University Hospital (Cádiz, Spain) throughout the year 2023 (1). NRP allows for the scheduling of automatic clinical trials for data collection, creating a folder structure for each trial where study metadata (study identifier, neonate ID, start time, end time, etc.) are stored, along with physiological data captured in real-time through an artificial vision system, positional data captured in real-time through an AI camera system, labeling data, and audio capture-related data (2).

NRP has two main cameras. The first camera is a regular camera (similar to a webcam) that focuses on the medical monitor connected to the neonate, where all vital signs (heart rate, SpO2, respiratory rate) are displayed. Through NRP, frames of the medical monitor’s front are captured, and using an algorithm called *CardMed*, which is based on computer vision (e.g., deep learning classification, image cropping, Optical Character Recognition, OCR ...), these physiological parameters are extracted from each frame along with a timestamp. This algorithm is extensively described in [8], where it was shown to have a reliability of 91% for heart rate and 90% for SpO2.

**Fig. 2:**
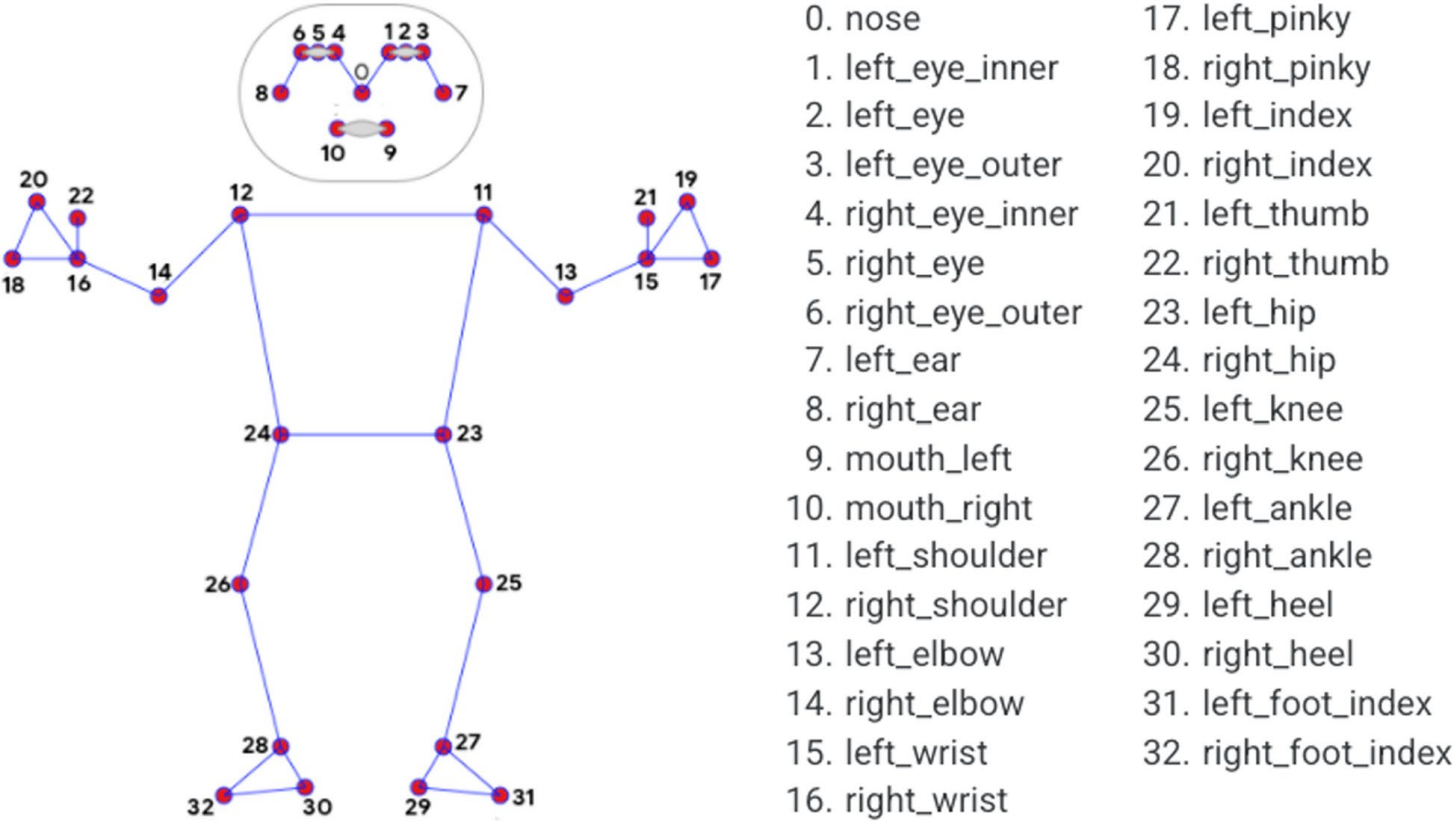
Pose landmarker model - Image obtained from (https://ai.google.dev/edge/mediapipe/solutions/vision/pose_landmarker) (Property of Google)

On the other hand, the second camera is a three-lens depth and AI camera (Luxonis OAK-D model^9^), which loads the pre-trained Google Mediapipe®model^10^ and allows the extraction of 33 body landmarks from the neonate in real time (>20 frames per second, fps). For each frame, all 33 landmarks are recorded in three dimensional spatial coordinates, that is, consisting of x, y, and z components (see Fig. 2). Additionally, when nurses or doctors access the incubator to handle the neonate, NRP only detects and stores the body of the neonate. On the other hand, if an arm, hand, or object obscures the body, the software does not store partial body data—only complete data—to ensure its quality. For this reason, all data recorded in NeoVault correspond to full-body captures of the neonate.

To this folder structure, a CSV file containing medical data known only to medical staff (for privacy reasons) is added (so far this process is done manually) (3). This entire information is compressed into a single file (4) that is used by NeoVault as input data. Currently, this information is uploaded through the RESTful API with security constraints (5), which is then transformed into database records via a parser implemented in Python.

Once the information is registered, it is easily and conveniently accessible through the web interface for both medical personnel or data scientists (6), as well as for programmers or developers using the API (7), aiming to integrate this information in real-time and automatically into other systems or solutions.

The Web interface is structured around four blocks: *(a)* filtering and searching for neonates; *(b)* list of available neonates along with medical information; defining a time frame for *(c)* postural information, where you can filter to indicate which parts of the body you want to include in the dataset; and finally, *(d)* physiological parameters (heart rate and oxygen saturation).

The search block *(a)* allows obtaining all available neonates (without filters) or applying a filter according to the following parameters: *1)* gender (boy, girl, both); *2)* gestational age (value between 25 and 36 weeks); *3)* birth size (value between 20 and 50 centimeters); *4)* birth head circumference (value between 15 and 45 centimeters); *5)* minimum and maximum birth weight (in grams); *6)* APGAR 1 and APGAR 5 scores (values between 0 and 10); *7)* CRIB (Clinical Risk Index for Babies) score (value between 0 and 23); and finally, whether the neonate has *8)* brain damage or not. Additionally, in this block, each parameter can be enabled or disabled to consider it in the search or not.

**Fig. 3:**
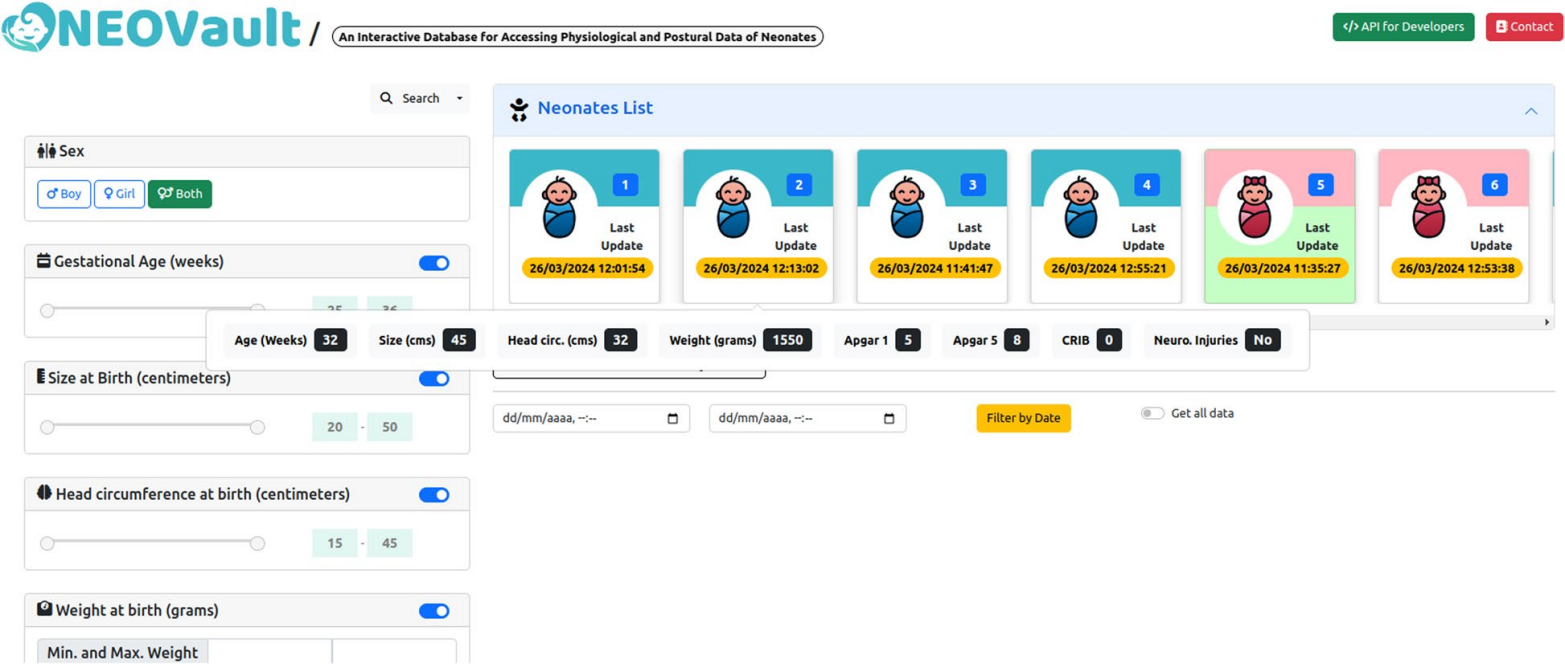
Search by medical parameters and neonate list in web interface

Once the search criteria are applied *(b)* , the available neonates are listed, displaying for each of them the gender (using blue color for male and pink for female, along with an illustrative icon), the neonate identifier (integer number), and the last data update date. Additionally, when hovering over each neonate, the values associated with the medical parameters indicated in the search block (*a*) are displayed in a pop-up. Figure 3 shows an illustrative example of these two blocks.

**Fig. 4:**
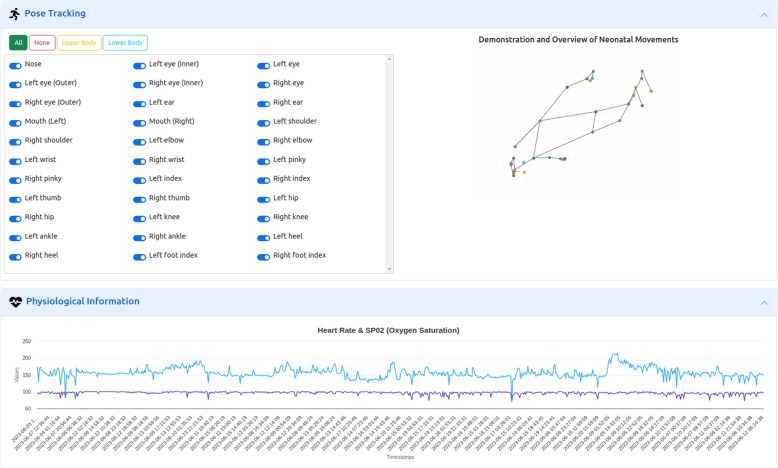
Postural and physiological data

From a selected neonate, it is possible to search for postural parameters data within a date range (*c*). NeoVault provides data for 33 landmarks (see footnote 5), and the platform allows selecting the upper body, lower body, entire body, or a customized selection. As an illustrative example, the user can view a 2D representation showing a few seconds of movement of the selected neonate. The same search request also returns existing physiological parameters (heart rate, oxygen saturation) (*d*). In NeoVault, each data record has, and will continue to have, an associated timestamp (in milliseconds), regardless of the type of data. This allows for the chronological recording of all data and its subsequent exploitation, as well as the ability to observe evolution over time. For this reason. Additionally, instead of specifying a date range, the web interface allows returning all existing data for the selected neonate. Figure 4 illustrates these two blocks.

In order to enable automatic, real-time data access and integration into third-party systems, NeoVault provides an API for programmers/developers. This API follows a RESTful philosophy, and its usage description can be found on the page accessible from the web interface or from its specification through OpenAPI. All information for interacting with the services is described using JSON to ensure access by any platform or software. The services offered by the API include *1)* listing neonates according to medical parameters; *2)* obtaining postural data; and *3)* obtaining physiological data for a neonate within a time range. The specification of these RESTful API endpoints can be found clicking in the green button located in the top-right corner with the text “API for Developers”.

Although the most common way to access information is through one of these two channels (Web interface or RESTful API), the database can be found in NeoVault data repository^11^, which will be periodically updated to provide the same information as both access interfaces.

## Utility and discussion

In contrast to other databases dedicated to preterm infants, as highlighted in “Background” section), NeoVault stands out by offering a multitude of advantages. These differentiating factors contribute to its uniqueness and enhanced utility in the domain of neonatal care and research. A summary of the comparison between NeoVault and other databases is included in Table 2.

**Table 2:** Comparison of preterm infants data-base

Features	BabyPose	MIAdataset	Preterm Clinical Network (PCN)	MMSdataset	NeoVault
**Intuitive Navigation**	Yes	Yes	No	Yes	Yes
**Responsive Design**	Yes	Yes	Yes	Yes	Yes
**3D visualization tool**	No	No	No	No	Yes
**Data Type**	12 limb-joint locations and depth images related to the movement of neonates	A timeline of 16 different states vector of a preterm infant	Data concerning the care of women at risk of preterm birth and registry of children born to women at risk(born prematurely or not)	Perinatal data-neonatal data	Perinatal data-neonatal data; physiological parameters and 33 reference points of the preterm body
**User-friendly interface**	No	No	Yes	No	Yes
**Accessibility**	Public	Restricted	Restricted	Public	Public
**Support for Complex Queries**	No	No	Yes	No	Yes
**Collaborative environment**	No	No	Yes	No	Yes
**Cost**	Free	Free	Free	Free	Free
** Ease Import/Export**	Yes	No	No	Yes	Yes

One of the main contributions of NeoVault is its operation as a publicly accessible database, that houses perinatal and neonatal data, encompassing crucial physiological parameters such as oxygen saturation and heart rate. Additionally, it meticulously provides spatial data of 33 landmarks defining the body pose of preterm infants, offering an understanding of their intricate neuromotor development. The platform goes beyond mere data storage by providing an useful 2D visualization tool of neonates body movements.

Moreover, NeoVault emerges as an invaluable resource for healthcare professionals, researchers, and AI scientists. In the realm of neonatal care, NeoVault could play a pivotal role in early diagnosis thanks to the data that can be found in the database, empowering healthcare practitioners to identify developmental deficits in the preterm infant’s stages. This early detection is crucial for the initiation of therapeutic interventions, fostering optimal outcomes for the neonates.

Although, in the AI era, NeoVault stands at the forefront, offering a robust foundation for training AI models. The data housed within the database becomes a training ground for cutting-edge AI, enabling the automation of early detection processes for neonatal medical issues. This intersection of healthcare and AI holds the promise of revolutionizing the efficiency and accuracy of neonatal care.

Beyond its substantive data holdings, NeoVault distinguishes itself with a user-friendly interface. This interface is not merely a gateway for data retrieval, also provides seamless data visualization, integration, and formatting, enhancing the overall user experience and making NeoVault a versatile and indispensable database in the landscape of preterm infants healthcare and research. On the other hand, the possibility of accessing NeoVault data through its RESTful API not only enables another way for developers or programmers to access the data, but also the opportunity to access that data in real-time and integrate such access into other applications or systems, such as hospital systems, private software, medical repositories for data scientists, etc.

The current version of NeoVault is stable, but it is true that there are several shortcomings which are being considered for future work and will be implemented in the near future. These planned improvements include:

1. *User management system*. Currently, information is uploaded manually without any record of which users (neonatologists, doctors) contribute that information. The aim is to add a user management system where each medical personnel can manage their neonates through a username/password access method. However, the user/password access method, while necessary, is not sufficient. Additional personnel verification mechanisms will be implemented to ensure that any user wishing to register to upload information is indeed healthcare or research personnel. For example, institutional email will be verified, and professional credentials or those from their institution will be requested, among other methods, to validate their identity and role.
2. *Improve the system for adding data*. Currently, adding new information is done through the RESTful API using a username and password to prevent unauthorized users from uploading false or unreliable data in the current version. With the user management mentioned in (1), each identified user will be able to upload data more easily and with more credibility. This means opening up the capability for the rest of the community to contribute new data. NeoVault accepts data structured according to the format used by *NRP*. In future versions, there are plans to publish the specification so that any researcher or medical personnel can adapt their data to the format accepted by NeoVault and upload it easily.
3. *Automatic data dump from NRP*. Although the option to upload information manually (2) will be available, the NRP software (used as data collector) [8] will be modified so that the data collected is automatically saved in NeoVault, eliminating the need for manual processing.
4. *Adding new data sources*. In addition to physiological and postural parameters, NRP records audio and labels made by medical personnel that can be included as data sources in NeoVault. Additionally, external data sources can be added to NeoVault, such as blood analysis results or medical test outcomes (e.g., heel prick test) and clinical data that may be useful from a medical standpoint, such as post-menstrual age, whether the birth was by cesarean section, premature apnea, systolic murmur or even maternal characteristics like genetic factors, age, pre-existing medical conditions like diabetes, hypertension, heart disease, etc.. These data will be added based on medical criteria and the usefulness they may have as fundamental variables for data exploitation. The different improvements will be gradually included in NeoVault.
5. *Automatic tagging of events*. An improvement of the recording of the data will be that of the implementation of algorithms for the automatic annotation of the videos. For example, an algorithm could be applied to the video source during recording to classify events happening in the field-of-view and to add tags such as if the baby is present or not, if the baby is being manipulated or not by the medical staff, or if the position of the baby is not in supine.
6. *Ensuring data quality*. Although all information stored in and uploaded to NeoVault is consistent, with no empty values, null values, etc., there remains the possibility—either due to error or ill intent from users in the future—that the data, despite following a correct structure, may not be accurate or reliable. This means that the data could be fabricated, generated (for example, through generative AI), erroneous, or duplicated. Therefore, in the future, work will focus on algorithms or techniques that allow for the analysis of movement patterns before uploading the data to determine if they are coherent and real, thus minimizing the risk of erroneous data contaminating the rest of the reliable dataset.

## Conclusions

NeoVault stands as a data hub for contributing to advancement in neonatal healthcare research. It is a platform created to become a key resource, offering researchers, medical professionals, students, and data scientists access to meticulously organized datasets of preterm infants. These datasets would not only facilitate investigations into diagnostic improvements and treatment strategies but also empower studies focused on early disease detection, providing valuable insights for enhancing neonatal care protocols.

At its core, NeoVault emerges as a publicly accessible database, offering a comprehensive repository of movement data, physiological parameters, and neonatal and perinatal records for preterm infants who underwent hospitalization in a NICU. Its user-friendly interface allows users to define and filter queries, visualize datasets, and seamlessly export results in various file formats. The platform goes beyond conventional data storage by providing an advanced 3D visualization tool, enabling users to explore and analyze neonatal movements dynamically.

Future initiatives involve leveraging the database for cutting-edge studies, particularly in the realm of early detection of neurological issues, such as those managed by the PARENT project^12^. Additionally, NeoVault envisions establishing connections with more extensive datasets encompassing larger populations of preterm infants, promising to broaden its scope and impact in the field of neonatal healthcare research.

## Data Availability

The data used is available through the web interface of the NeoVault platform ( https://conversational.ugr.es/neovault) as well as through the RESTful API https://conversational.ugr.es/neovault/api/v1. The raw dataset is available in the repository https://github.com/bihut/neovault-database.

## Abbreviations

AI: Artificial Intelligence
APGAR: Appearance - Pulse - Grimace - Activity - Respiration
API: Application Programming Interface
CRIB: Clinical Risk Index For Babies
CSS: Cascading Style Sheets
CSV: Comma-separated values
ECG: Electrocardiogram
GIF: Graphics Interchange Format
HTML: Hypertext Markup Language
JSON: Javascript Object Notation
My: co-founder Michael Widenius’s daughter name
NICU: Neonatal Intensive Care Unit
NIPS: Neonatal Infant Pain Scale
NRP: Neonate Recording Platform
OFC: Occipitofrontal Circumference
PARENT: PrematuRe Newborn Motor and Cognitive Impairments
PCN: Preterm Clinical Network
REST: Representational State Transfer
SOA: Service-Oriented Architecture
SQL: Structured Query Language

## Footnotes

1 https://flask.palletsprojects.com/en/3.0.x/

2 https://pandas.pydata.org/

3 https://numpy.org/

4 https://www.sqlalchemy.org/

5 https://developers.google.com/mediapipe/solutions/vision/pose_landmarker

6 https://getbootstrap.com/

7 https://jquery.com/

8 https://www.highcharts.com/

9 https://shop.luxonis.com/products/oak-d

10 https://ai.google.dev/edge/mediapipe/solutions/vision/pose_landmarker

11 https://github.com/bihut/neovault-database

12 https://parenth2020.com/
